# Lacunary polyoxometalate nanoclusters as versatile interface modifiers for perovskite solar cells

**DOI:** 10.1038/s41598-026-46107-7

**Published:** 2026-09-01

**Authors:** Apostolis Verykios, Yasemin Torlak, Fangwen Cheng, Mélissa Crespin, Marinos Tountas, Nikolaos Tzoganakis, Dimitrios K. Giannopoulos, Antigoni Cheilari, Georgios C. Vougioukalakis, Ermioni Polydorou, Zoi Georgiopoulou, Georgios Chatzigiannakis, Ioannis G. Aviziotis, Vassilis Psycharis, Efi-Maria Papia, Vassilios Constantoudis, Anastasia Soultati, Mahmut Kus, Mustafa Ersoz, Emmanuel Kymakis, Alexander Chroneos, Ioannis Karatasios, Feng Gao, Feng Wang, Maria Vasilopoulou

**Affiliations:** 1 Institute of Nanoscience and Nanotechnology, National Center for Scientific Research Demokritos, Agia Paraskevi, Attiki, 15310 Greece; 2 Cal Vocational High School, Pamukkale University, Denizli, 20700 Turkey; 3 Department of Physics, Chemistry and Biology, Linköping University, Linköping, 58183 Sweden; 4 Department of Electrical & Computer Engineering, Hellenic Mediterranean University, Estavromenos, Heraklion, GR-71410 Crete Greece; 5 Laboratory of Organic Chemistry, Department of Chemistry, National and Kapodistrian University of Athens, Panepistimiopolis, Athens, 15771 Greece; 6 School of Chemical Engineering, National Technical University of Athens, Heroon Polytechneiou 9, Zografou Campus 15771, Athens, Greece; 7 Department of Chemical Engineering, Konya Technical University, Konya, 42075 Turkey; 8 Department of Chemistry, Selcuk University, Konya, 42250 Turkey; 9 Department of Electrical and Computer Engineering, University of Thessaly, Volos, 38221 Greece; 10 Department of Materials, Imperial College London, London, SW7 2AZ UK

**Keywords:** Energy science and technology, Materials science, Nanoscience and technology, Physics

## Abstract

We report the first incorporation of diverse lacunary polyoxometalate (POM) nanocluster materials as efficient interface modifiers in perovskite solar cells (PSCs). Devices utilizing POM-modified SnO_2_ electron transport layers demonstrated marked improvements in open-circuit voltage (V_OC_), short-circuit current density (J_SC_), and fill factor (FF), achieving power conversion efficiencies exceeding 21.6%. Transient photovoltage and photocurrent analyses revealed that the B1-type based device exhibited the longest carrier lifetime and most rapid charge extraction rate, indicating optimized charge transport and suppressed recombination losses. Stability assessments under ambient conditions confirmed that B1-type POM-modified devices retained higher efficiency over time with reduced hysteresis compared to control devices. Work function measurements indicated POM-induced energy level alignment shifts, facilitating efficient charge transport. Morphological and structural analyses via scanning electron microscopy and X-ray diffraction confirmed that POMs using tungsten (W) addenda (i.e., B1–W and B2–W) promoted superior perovskite crystallization, yielding larger grain sizes and enhanced crystallinity. These findings establish the potential for lacunary POMs to serve as effective interface modifiers for advancing the efficiency and durability of perovskite-based optoelectronic devices.

**Supplementary Information:**

The online version contains supplementary material available at https://doi.org/10.1038/s41598-026-46107-7.

## Introduction

The pursuit of efficient and stable photovoltaic technologies has accelerated over the past decade, with solar energy emerging as a leading renewable energy source due to its sustainability and environmental benefits. Among various solar technologies, perovskite solar cells (PSCs) have garnered considerable attention for their exceptional potential in both efficiency and cost-effectiveness. Since their inception in 2009^1^ the power conversion efficiency (PCE) of PSCs has rapidly increased, reaching values over 25%^2^ positioning them as the frontrunners among third-generation photovoltaics. With the intense ongoing research efforts of scientists, certified single-junction perovskite solar cells (PSCs) now achieve an impressive power conversion efficiency (PCE) of 26.7%^2^, marking the beginning of PSCs’ industrial development. However, challenges related to device stability and interfacial losses continue to hinder their large-scale deployment^3–13^.

Recent research has focused on the integration of functional interlayers to address these limitations. One of the critical layers is the electron extraction layer (EEL), which is responsible for efficiently collecting and transporting the photogenerated electrons from the perovskite active layer to the external circuit. An ideal EEL should combine high electron mobility, excellent optical transparency, and good energy level alignment to minimize recombination losses and maximize power conversion efficiency (PCE)^14–17^. Amid different materials investigated, tin oxide (SnO_2_) has attracted significant interest as highly promising material for this role due to its thermodynamic stability under ambient conditions, superior electron transport properties, low optical absorption in the visible spectrum and adjustable direct band gap. Numerous studies have been published on enhancing the efficiency of photovoltaic devices by adjusting the energy band of SnO_2_ to align with that of perovskite.^18–20^ Compared to conventional electron transport layers such as titanium dioxide (TiO_2_), SnO_2_ provides a lower conduction band offset with perovskites, reducing energy barriers for charge extraction while simultaneously mitigating photocatalytic degradation under ultraviolet (UV) exposure, a known issue in TiO_2_-based PSCs. Thus, its favorable properties have made SnO_2_ the preferred material for electron extraction in many high-performance PSCs.

Tin oxide can be synthesized and deposited using various methods, including solution processing (e.g. spin-coating, spray pyrolysis),^21,22^ atomic layer deposition (ALD),^23^ and magnetron sputtering^24^. Among these, solution-processed SnO_2_ is widely favored for its low-temperature fabrication, affordability, and compatibility with flexible substrates.^21,25–27^ Nevertheless, SnO_2_ films often suffer from oxygen vacancies, surface defects, and charge traps that can function as recombination centers, adversely affecting device performance and long-term stability.^28^ Consequently, researchers have explored numerous strategies to optimize SnO_2_-based EELs using elemental doping,^29–35^ incorporating ionic compounds^36–41^, carbon-based materials^42–44^, organic molecules^45–51^ and metal oxide layers^52–54^.

Polyoxometalates (POMs), on the other hand, represent a vast and diverse class of inorganic nanoclusters with relatively rigid structure composed of early-transition metal-oxygen anion networks. These nanoclusters are synthesized using both conventional and soft experimental pathways.^55,56^ POMs are distinguished not only for their multitudinous architectures but also for their exceptional properties in terms of high solubility in water-alcohol mixtures^57^, remarkable transparency in the visible spectrum^58^ and favorable electron mobility^59^, which collectively enhance their versatility in various applications, such as catalysis^60^, energy conversion^61^ and molecular electronics^62^. POMs with either the Keggin or Dawson structural motifs have been employed as hole or electron injection/extraction layers, tailored according to their specific optoelectronic properties^63,64^. In addition, our group has shown that the lacunary Keggin-type POMs - specifically potassium sodium 11-tungstenphosphate (α-K_7–x_Na_x_PW_11_O_39_·14H_2_O, referred to as B1–W) and potassium sodium 11-molybdophosphate (α-K_7–x_Na_x_PMo_11_O_39_·14H_2_O, referred to as B1–Mo) - can be effectively applied as electron injection materials in both fluorescent and phosphorescent organic light-emitting diodes (OLEDs)^65^. Later on, the same POMs along with potassium 9-tungstenphosphate (α-K_9_PW_9_O_34_·16H_2_O, termed B2–W) were successfully incorporated as electron interlayers in cathode of inverted polymer solar cells^66^. These benefits underscore lacunary POMs as strategic materials for advancing optoelectronic device efficiency, durability, and scalability. However, their application to perovskite devices has not reported up to date.

In this work, we report the first application of the lacunary POMs B1-Mo, B1-W, and B2-W, as well as the potassium sodium 9-molybdophosphate (α-K_7–x_Na_x_PMo_9_O_34_•16H_2_O, referred to as B2–Mo), as electron interface modifiers between SnO_2_ and the perovskite absorber. The key difference between the B1- and B2-type POMs lies in their structure and charge, which are influenced by the number of missing M(W, Mo)O_6_ octahedra. B1-type POM possess a negative charge of -7 due to the removal of one M(W, Mo)O_6_ unit, while B2-type POM has a higher negative charge of -9, as it lacks three WO_6_ octahedra. We elucidate the role of POM structure and their impact on SnO_2_/perovskite interface modification as well as on the photovoltaic performance and stability of PSCs by analyzing charge carrier dynamics, device efficiency, and long-term stability among other factors. We demonstrate that POMs not only enhance charge extraction but also significantly improve the crystallization and morphology of perovskite films. These findings suggest that POM modification of SnO_2_ interlayers offers a promising strategy for advancing the efficiency and durability of PSCs, providing a path toward the commercialization of perovskite-based optoelectronic devices.

## Results

### Device characterization and stability

**Fig. 1:**
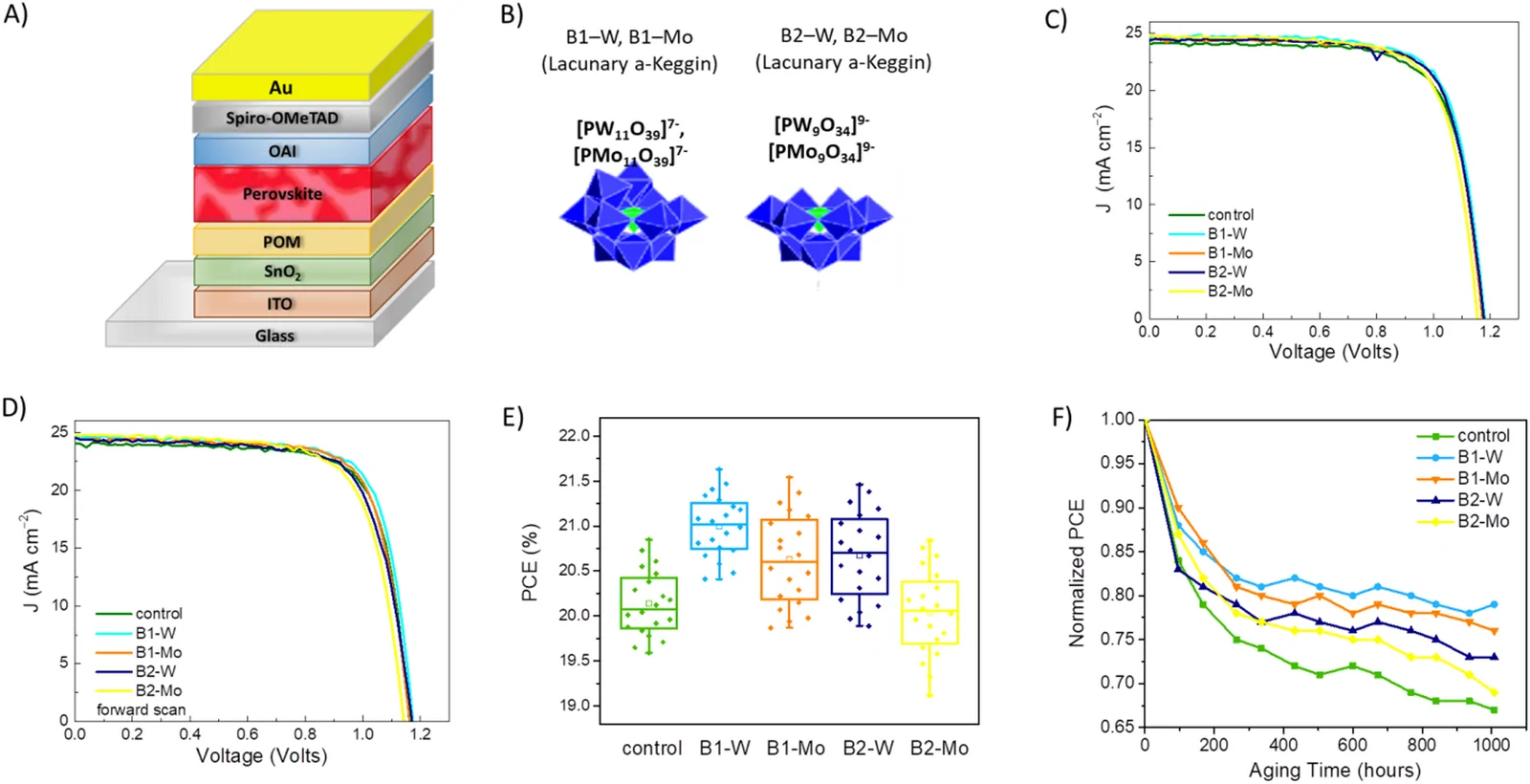
(**A**) Device structure of the POM-modified perovskite solar cells. (**B**) The chemical structure of POMs. J-V curves of control and POM-based perovskite solar cells scanned in (**C**) reverse and **D**) forward directions. (**E**) Box plots of the perovskite solar cell PCE with different EELs. (**F**) Variation of normalized PCE upon aging over a period of 1000 hours using as-deposited SnO_2_ and POM-modified SnO_2_ interlayers.

To unravel the impact of POM interlayers on PSCs, the photovoltaic performance of control and POM-modified devices was characterized using the device configuration glass/ITO/SnO₂ or POM-modified SnO₂/perovskite/OAI/spiro-OMeTAD/Au (where ITO represents indium tin oxide, SnO_2_ refers to tin oxide, OAI represents n-octylammonium iodide and spiro-OMeTAD represents 2,2′,7,7′-tetrakis(N, N-di-p-methoxyphenyl-amine)9,9′-spirobifluorene), where the perovskite is formamidinium lead iodide (FAPbI_3_, see experimental section). Figure 1A presents the schematic illustration of the device structure and Fig. 1B shows of the chemical structure of POMs used in this study exhibiting the lacunary a-Keggin structure. The current density–voltage (J–V) characteristic curves of the control (without the POMs) and the POM-modified devices, as measured during a reverse and forward scan are shown in Fig. 1C and D, respectively. The photovoltaic parameters of all the devices are summarized in Table 1 and the statistical distribution of the perovskite solar cells PCE with and without POMs is also presented in Fig. 1E. According to Table 1, it becomes evident that the devices based on B1- and B2-modified SnO_2_ interlayers showed improvement in their open circuit voltage (V_OC_), short circuit current (J_SC_) and fill factor (FF). In particular, the control device with the as-deposited SnO_2_ exhibited a relatively wide power conversion efficiency (PCE) distribution with maximum PCE of 20.74% having a V_OC_ of 1.173 V, a J_SC_ of 24.14 mA cm^-2^ and a FF of 73.26%. On the other hand, B1–W and B2–W based PSCs yielded maximum PCE values of 21.63% and 21.46%, respectively, with V_OC_ values of 1.181 V and 1.177 V, J_SC_ values of 24.76 mA cm^-2^ and 24.47 mA cm^-2^, and fill factors (FF) of 74.00% and 74.52%, respectively. Although the devices incorporating B1–Mo- and B2–Mo-modified SnO_2_ layers demonstrated enhanced performance, their improved PCEs were attributed mainly to the increase of the photocurrent (24.68 mA cm^-2^ for B1–Mo and 24.89 mA cm^-2^ for B2–Mo). The PCE distribution of B1-W is significant narrower compared to the rest of devices demonstrating the more reliable and stable device performance. Furthermore, the photocurrent hysteresis phenomenon in the control and the POM-modified devices was evaluated. B1-W based device presented a reduction in the current hysteresis (0.369%) compared with that of the control device (0.576%), indicating stable electrical characteristics. Β2–W-based device exhibits a significantly higher hysteresis value (3.875%), leading to a difference between the PCE of 21.46% (reverse scan) and the PCE of 20.59% (forward scan) and thus, suggesting some instability in its performance. B1–Mo- and B2–Mo-based PSCs also show noticeable hysteresis values, with B1–Mo at 1.275% and B2–Mo at 3.368%, denoting that while these devices perform better in terms of PCE, they exhibit wider PCE distribution and thus higher instability compared to the control and B1-based devices.

Transient photovoltage (TPV) and transient photocurrent (TPC) decay measurements presented in Figure S1A and B, respectively, provide insights into charge carrier dynamics and recombination processes in the PSCs incorporating different POM-modified SnO_2_ interlayers. The TPV decay (Figure S1A) illustrates the recombination dynamics, where a slower decay corresponds to a longer charge carrier lifetime and reduced non-radiative recombination. The fastest decay is observed for B2–Mo, indicating the shortest carrier lifetime and highest recombination losses. The control device exhibits a slightly slower decay, suggesting that unmodified SnO_2_ does not effectively suppress recombination. The B2–W and B1–Mo samples show similar intermediate decay rates, indicating moderate recombination suppression. The B1–W-modified device demonstrates the slowest decay, signifying the longest carrier lifetime and the most effective suppression of recombination. The TPC decay (Figure S1B) reflects charge extraction efficiency, where a faster decay indicates improved carrier transport and collection. The B1–W-modified device exhibits the fastest decay, followed closely by B1–Mo, suggesting that B1-type modifications enhance charge extraction. B2–W shows a slightly slower decay, indicating slightly lower extraction efficiency.

The control device exhibits a further slowed decay, highlighting its inferior charge collection. Notably, B2–Mo, despite exhibiting the highest J_SC_, has the slowest TPC decay, implying that while it generates a high number of charge carriers, transport limitations or recombination losses hinder efficient extraction. This observation aligns with its relatively lower FF and PCE in the case of B2–Mo, suggesting that the excess carriers may recombine before being collected. Moreover, the extended carrier lifetime in B1-modified devices corresponds with their enhanced V_OC_, FF and thus PCE, while the improved charge extraction in B1–W and B1–Mo suggests enhanced current density. In contrast, the high recombination rates observed for B2–Mo and its slower charge extraction may explain its lower stability and increased hysteresis. The modification of SnO_2_ with the POMs, and especially with the B1-family improved also the lifetime time of the corresponding PSCs. For the stability studies of the prepared PSCs, the fabricated devices were stored in a N_2_-filled glovebox without encapsulation, and their performance was measured at ambient conditions for 1,000 h. The normalized PCE values of the devices are plotted as a function of the store time, as shown in Fig. 1F. During the aging time of 1,000 h, the POM-modified SnO_2_ PSCs were more stable in respect with the control device. Moreover, a significant degradation of the control device performance was observed, maintaining only 67% of its initial PCE after 1,000 h. Similarly, the B2–W and B2–Mo-modified devices exhibited intermediate performance stability, reaching ~ 73% and ~ 69% retention, respectively, underscoring their limited stability. This instability may be attributed to the higher hysteresis, as well as the higher recombination losses as evident by the slower TPC decay of the POM-B2 group, compared with the POM-B1 family (Figure S1B). On the other hand, B1–W-modified device demonstrated enhanced durability maintaining the 79% of its initial PCE value after 1,000 h, reflecting a 21% degradation. Lastly, the B1–Mo-modified device exhibited a trend similar to that of the B1–W device exhibiting a PCE degradation of 24%. The results suggest that while all devices undergo efficiency loss over time, the B1–W-based PSC exhibiting improved charge extraction, reduced non-radiative recombination and decreased hysteresis maintains superior stability, compared with the fast degradation of the control device.

**Table 1:** Performance characteristics of perovskite solar cells with the device configuration Glass/ITO/SnO₂ or POM-modified SnO₂/perovskite/OAI/spiro-OMeTAD/Au, as measured in reverse and forward scan.

Device	Scan detection	V_OC_ (V)	J_SC_ (mA/cm^2^)	PCE (%)	FF (%)	Hysteresis (%)
Control	Reverse	1.173	24.14	20.74	73.26	0.576
	Forward	1.162	24.16	20.85	74.28	
B1–W	Reverse	1.181	24.76	21.63	74.00	0.369
	Forward	1.170	24.70	21.49	74.37	
B2–W	Reverse	1.177	24.47	21.46	74.52	3.875
	Forward	1.171	24.58	20.59	71.54	
B1–Mo	Reverse	1.172	24.68	21.54	74.47	1.275
	Forward	1.162	24.72	21.22	73.86	
B2–Mo	Reverse	1.155	24.89	20.84	72.49	3.368
	Forward	1.142	24.86	20.14	70.95	

To further investigate the role of POMs as interlayers in effectively passivating SnO_2_ and thereby improving device performance—and to exclude the possibility that the DI water solvent itself contributes to the passivation effect—SnO_2_/DI-treated/PVSK-based device was also fabricated. Figure S2 presents the J–V characteristic curves of the SnO_2_/DI-treated/PVSK-based PSCs in comparison with the control device and the best-performing B1-W-based devices. No significant difference in current density is observed between the control and DI-treated SnO_2_ devices, indicating that DI water treatment alone does not contribute to performance enhancement. On the other hand, the PSC incorporating the POM B1-W exhibits an increased current density, confirming the beneficial role of POM as an effective passivator of SnO_2_.

### Surface work-function (W_F_) measurements

**Fig. 2:**
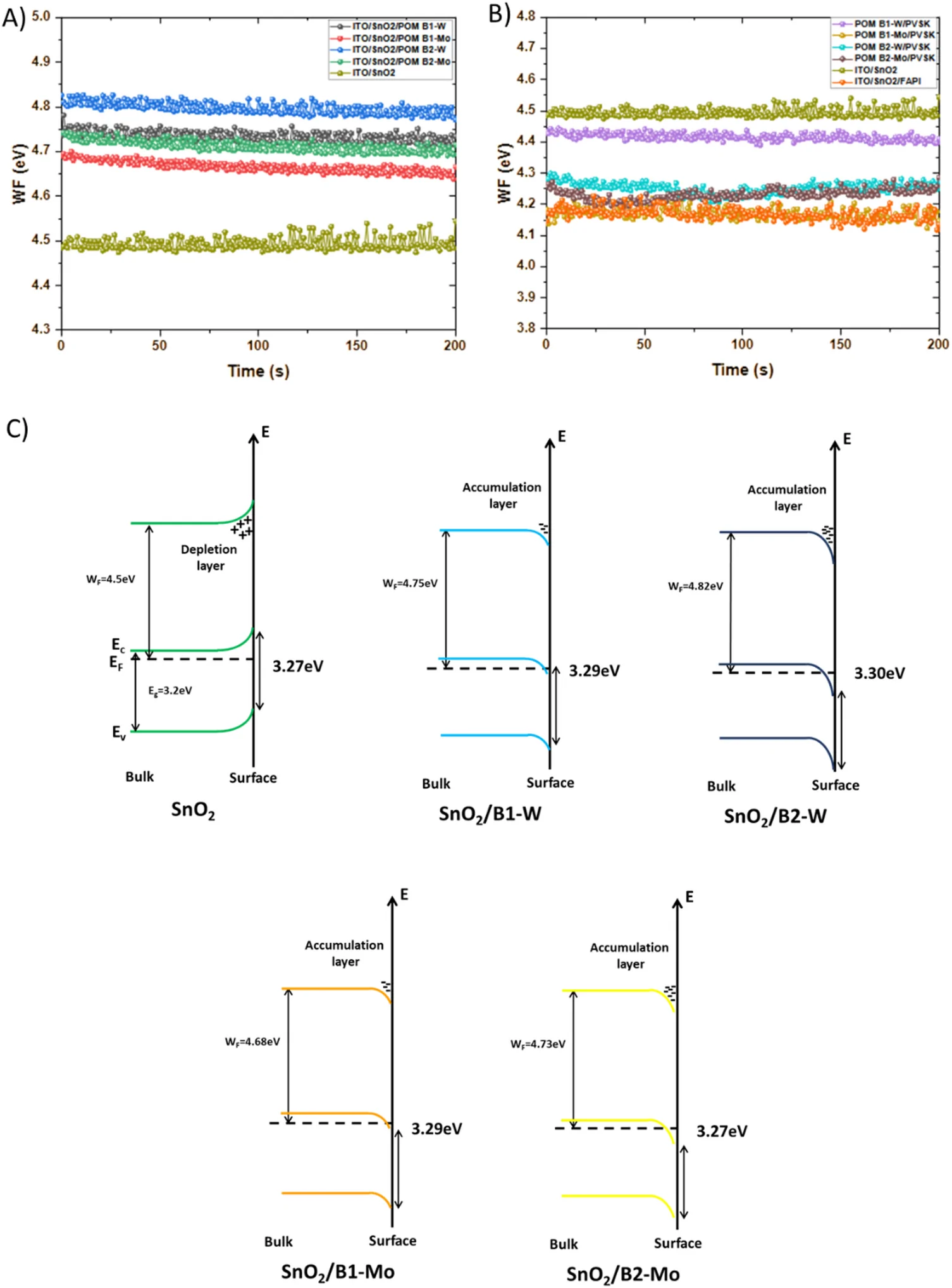
Surface work function (W_F_) measurements of ITO/SnO₂-based interfaces with and without POM modification, (**A**) before and (**B**) after perovskite deposition. (**C**) Schematic representation of band diagrams of the pristine and POM-modified SnO_2_ layers.

The surface work function (W_F_) of various SnO_2_-modified films deposited on ITO substrates was systematically investigated to assess the impact of POM modification. The measured surface W_F_ values for POM-modified SnO_2_ compared with the pristine SnO_2_ are shown in Fig. 2A and summarized in Table 2.

The unmodified SnO₂ substrate exhibits a W_F_ of 4.50 eV. Upon deposition of POM interlayers, the W_F_ systematically increases, with values ranging from 4.68 eV to 4.82 eV depending on the POM composition. Specifically, B1 POMs give W_F_ values of 4.75 eV (B1–W) and 4.68 eV (B1–Mo), while B2 POMs result in a more pronounced shift, with B2–W reaching 4.82 eV and B2–Mo at 4.73 eV. It should be noted that the absorption of POMs in the UV range is attributed to ligand-to-metal charge transfer (LMCT) and the electronic transition within the metal-oxygen framework of the POMs, thus the differences in absorption intensity among the POMs (Figure S3) suggest variations in their electronic density and charge delocalization, which can impact their ability to modify the SnO_2_ work function. These results indicate that B2-type POMs induce higher W_F_ compared to B1-type counterparts, with tungsten-based POMs (W) exhibiting a stronger effect than molybdenum-based POMs (Mo). The increase in W_F_ suggests enhanced electron selectivity at the interface, which could facilitate charge extraction while reducing recombination losses^67^ leading to improved device efficiency and stability, as shown in Fig. 1E and F. One possible mechanism is through chemical passivation, where the dangling bonds with hydroxyl groups (–OH) bound to Sn^4+^ present at the surface of SnO_2_ are terminated. Note that hydroxyl groups are originated by the water (solvent) of SnO_2_ solution. In addition, fixed negative charge carriers formed on the POMs surface reduces the depletion layer of the pristine, non-passivated SnO_2_ surface leading to a downward band bending, as illustrated in Fig. 2C causing a second passivation mechanism called “field-effect passivation”. In this case, a surface accumulation layer is formed which is more pronounced in the B2-type POMs. It is noted that the energy gap of the pristine and POM-modified SnO_2_ films were estimated from the Tauc plots shown in Figure S4A as derived from the absorbance spectra (Figure S4B).

**Table 2:** Summary of surface work function (W_F_) values of various ITO/POM-modified SnO_2_ films compared with the unmodified ITO/SnO_2_ film, and of perovskite (PVSK) films deposited on the pristine and POM-modified SnO_2_ substrates.

Sample	W_F_ (eV)
ITO/SnO₂	4.50
ITO/SnO₂/POM B1–W	4.75
ITO/SnO₂/POM B1–Mo	4.68
ITO/SnO₂/POM B2–W	4.82
ITO/SnO₂/POM B2–Mo	4.73
ITO/SnO_2_/PVSK	4.14
ITO/SnO_2_/POM B1–W/PVSK	4.44
ITO/SnO_2_/POM B1–Mo/PVSK	4.18
ITO/SnO_2_/POM B2–W/PVSK	4.25
ITO/SnO_2_/POM B2–Mo/PVSK	4.22

The surface W_F_ of the perovskite film deposited on top of pristine and POM-modified SnO_2_ was also investigated (Fig. 2B; Table 2). The ITO/SnO_2_/PVSK sample exhibited a W_F_ of 4.14 eV, while the introduction of POM interlayers significant increases the W_F_ of perovskite deposited on top, depending on the specific POM. Particularly, the B1–W/PVSK and B1–Mo/PVSK configurations exhibit W_F_ values of 4.44 eV and 4.18 eV, respectively, while B2–W/PVSK and B2–Mo/PVSK result in W_F_ values of 4.25 eV and 4.22 eV, respectively. The correlation between W_F_ and device performance reveals that higher W_F_ values align with increased open-circuit voltage (V_OC_) and reduced hysteresis. While B2-type POMs induce the largest W_F_ increase, they also exhibit higher hysteresis and faster degradation, suggesting that excessive W_F_ shifts may hinder charge extraction or introduce instability at the interface. On the other hand, B1-W provides an optimal balance, increasing the W_F_ while maintaining device stability. These results highlight the importance of controlled W_F_ modulation in optimizing charge transport and device longevity in perovskite-based optoelectronic devices. By tuning the interfacial energy alignment via molecular interlayers like POMs, charge carrier extraction was improved and non-radiative recombination was reduced at the interface between the POM-modified SnO_2_ and perovskite layer, resulted in higher performance.

### Morphological and structural analysis of perovskite films deposited on POM-modified SnO_2_ layers

The atomic force microscopy (AFM) images (Figure S5 and S6) provide insights into the surface morphology of perovskite films deposited on different POM-modified SnO_2_ substrates. The root mean square roughness (R_rms_) values reflect variations in film uniformity, grain distribution, and interfacial contact, which are critical for charge transport and device stability. The control sample exhibits the highest roughness (R_rms_ = 37.46 nm), indicating non-uniform grain growth that can increase recombination losses. This is in line with the TPV and TPC data, where the control device shows faster voltage decay and slower charge extraction, suggesting inefficient carrier transport and faster device degradation. B1-modified surfaces show reduced roughness (B1–W: 33.96 nm, B1–Mo: 34.81 nm), indicating more uniform perovskite crystallization. The improved film morphology enhances interfacial contact and charge transport, consistent with the faster TPC decay observed in B1–W, which also correlates with its higher fill factor (74.37%) and thus, improved power conversion efficiency (21.63%). B2-modified surfaces exhibit mixed trends, with B2–W (33.33 nm) being the smoothest but still showing higher recombination, while B2–Mo (35.96 nm) has increased roughness, leading to greater non-radiative recombination, lower fill factor (70.95%), increased hysteresis, and thus higher instability compared with the B1-W-based PSC. To have a deeper understanding on how POM modifications influence the grain structure and boundary characteristics of the overlying perovskite, the watershed-based and correlation length methods were conducted. According to watershed-based method (Figure S7A), the boundary-to-whole area ratio for the films was measured, showing that B2–W-modified surface has the largest grain boundary length, then B2–Mo followed by B1-modified surfaces, with the control film having the smallest boundary length. A larger grain boundary length indicates a larger grain density and more surface defects (Figure S7). This correlates with the higher roughness observed in the AFM analysis for B2-modified films, suggesting that the latter introduce more grain boundaries, leading to increased recombination sites. This explains the moderate performance, increased hysteresis and fast degradation of the B2-modified devices. Then, the correlation length for each sample was estimated (Figure S7B, Table 3), with B2-W having the smallest correlation length (172 nm), indicating smaller grains compared to the other samples.

**Table 3:** Correlation lengths of perovskite films deposited on pristine and POM-modified SnO_2_ substrates.

Image	Correlation length (nm)
Control	193
B1–W	185
B2–W	172
B1–Mo	186
B2–Mo	186

On the other hand, the control sample has the largest correlation length (193 nm), implying that it has relatively larger grains compared to the POM-modified films. The fact that the control film has larger grains and lower boundary lengths (AFM images and correlation length) would typically suggest fewer recombination sites. However, the rougher surface (AFM) and unmodified interfaces contribute to higher recombination rates and inferior performance, which aligns with its relatively lower photovoltaic performance compared to POM-modified films. These results suggest that POM interlayers influence perovskite crystallization, with B1-based modifications producing smoother, more uniform films that improve charge transport and reduce recombination. While B2-modified films also improve morphology, their higher recombination rates suggest less favorable interfacial properties. This reinforces that B1-based POMs provide the best conditions for enhanced photovoltaic performance and stability.

**Fig. 3:**
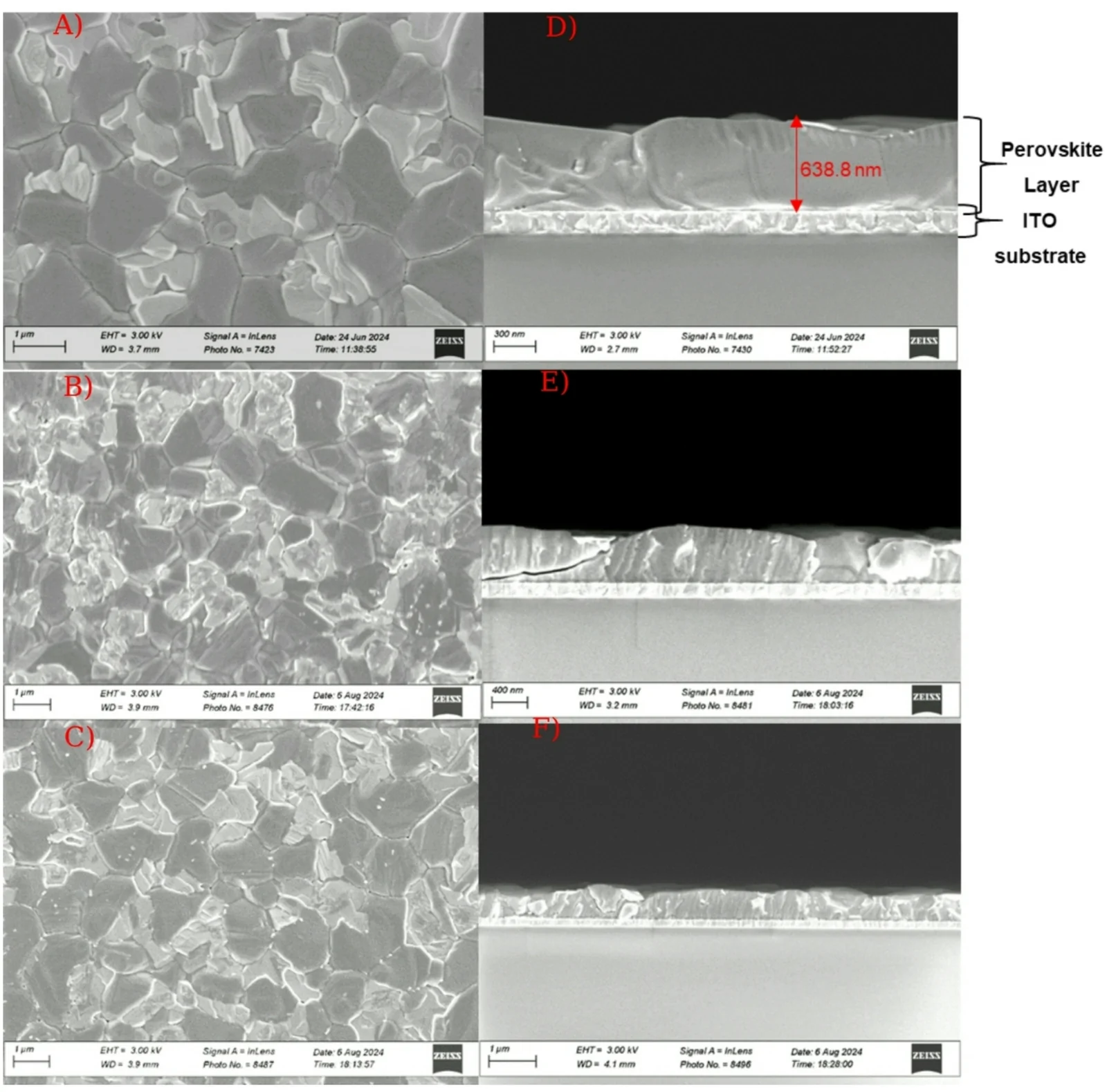
Surface and cross-sectional SEM images of perovskite films deposited on the SnO_2_ substrates with B1–W (**A**, **D**), B2–W (**B**, **E**), or no POM modification (**C**, **F**).

While all POM-modified SnO₂ layers showed performance enhancements, B1–W- and B2–W-based devices demonstrated the most significant improvements in photovoltaic efficiency. Given these advantages, a deeper investigation into their impact on perovskite film morphology and crystallization was conducted, using scanning electron microscopy (SEM) and X-ray diffraction (XRD) to further elucidate their role in optimizing interfacial properties. By increasing the efficiency of the devices, the effect of the POMs on the crystallization process of perovskite is expected, which should lead to bigger grains size. Indeed, larger grains mean fewer grain boundaries in the material. Grain boundaries are areas where defects can form, leading to non-radiative recombination of charge carriers, which could lead to poor charge collection. In addition, the reduction of grain boundaries could lead to an easier mobility of the charges and consequently, to a better extraction. Figure 3C, A and B shows the top view SEM images of perovskite films deposited on SnO_2_, SnO_2_/B1-W, and SnO_2_/B2-W, respectively, revealing the formation of uniform films in all cases.

Moreover, the cross sections SEM images (Fig. 3D, E and F) show that the thickness of the perovskite films is slightly affected by the underlying layer. More importantly, based on Table 4, the perovskite films deposited on B1–W and B2–W exhibit higher grain size than the control perovskite film, spin-coated on SnO_2_, leading to improved V_OC_ values, and thus, higher device performance compared with the control PSC.

To further investigate the effect of the POMs solvent (DI water) on the morphology of the perovskite films, SEM measurements were also conducted on DI water–treated SnO_2_/PVSK sample and compared with the SnO_2_/PVSK and the best-performing SnO_2_/B1-W/PVSK films. The corresponding the top-view SEM images of perovskite films deposited on pristine, DI water-treated, and B1-W-modified SnO_2_ substrates are presented in Figure S8. No significant differences are observed between the two films (Figure S8A and B, respectively), indicating that the DI water surface treatment of SnO_2_ does not noticeably influence the morphology of the subsequently spin-coated perovskite layer. On the other hand, the SEM image of the B1-W-based sample (Figure S8C and Fig. 3A) reveals a more compact and tighty packed grain structure compared with the control and DI water-treated samples. The grains appeare better interconnected suggesting improved film coverage and enhanced grain boundary cohesion, which may contribute to suppressed defect density and more efficient charge transport across the perovksite film. Furthermore, the more uniform grain distribution and larger grains indicates that the incorporation of B1-W favors improved crystallization and film formation dynamics.Therefore, the variations in the PSCs performance are unlikely to originate from morphological differences in the perovskite film upon DI-water treatment of SnO_2_ EEL, but rather from interfacial modification effects induced by the insertion of POMs in the device.

**Table 4:** Average grain sizes and thicknesses of perovskite films deposited on pristine, B1-W- and B2-W-modified SnO_2_ substrates.

Samples	Average grain size (µm)	Thickness (nm)
B1-W	1.879	638.8
B2–W	1.692	565.4
Control	1.401	587.9

**Fig. 4:**
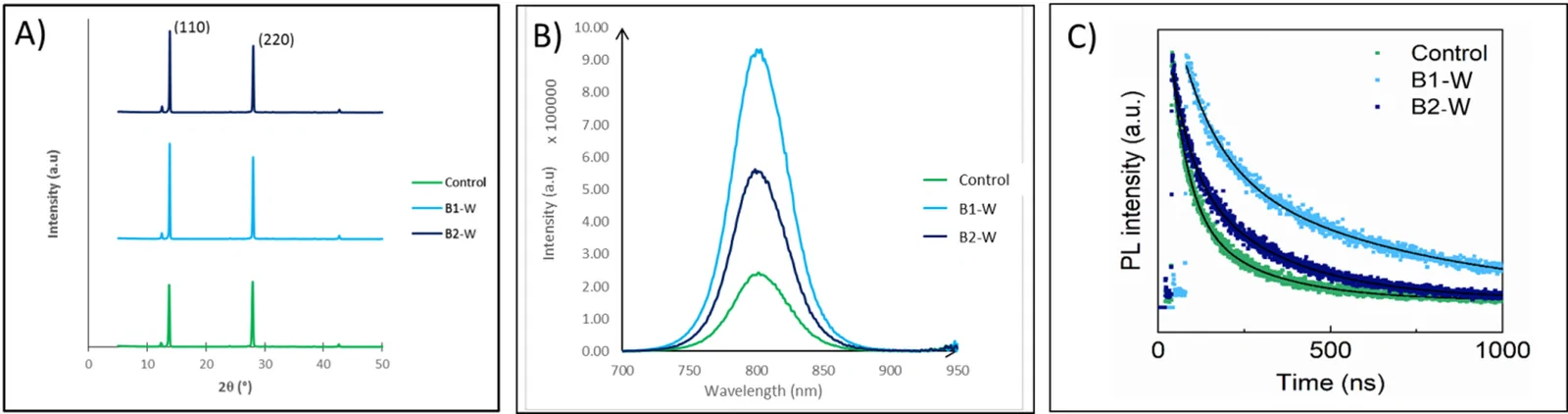
(**A**) XRD diagrams, (**B**) PL, and (**C**) TRPL spectra of the perovskite films deposited on pristine (control) and B1-W and B2-W-modified SnO_2_ substrates.

Furthermore, the crystallinity of the perovskite films deposited on SnO_2_, SnO_2_/B1-W, and SnO_2_/B2-W was studied using XRD measurements. XRD analysis can identify the different phases present in the perovskite layer, and therefore detect whether the incorporation of POMs has influenced the formation of the perovskite phase or introduce new phases. The XRD patterns of the SnO_2_/perovskite, SnO_2_/B1-W/perovskite, and SnO_2_/B2-W/perovskite are presented in Fig. 4A. Figure S9 shows also the XRD pattern of the perovskite film deposited on DI water treated-SnO_2_ for comparison reasons. All samples show the same crystallinity peaks with no new phases created due to the incorporation of POMs. Moreover, the comparable XRD peak intensities for both the control sample and the perovskite film spin-coated on DI-water treated-SnO_2_ suggest a similar degree of crystallinity. On the other hand, the XRD peaks of the perovskite films deposited on B1–W or B2–W (Fig. 4A) have higher intensity, suggesting improved crystallinity. In accordance to SEM analysis and the increase of the grain size, this crystallinity enhancement resulted in higher electrical parameters for the PSC based on B1-W and B2-W, and thus higher PCE values compared with the control device.

### Interface recombination and charge transfer with photoluminescence (PL) analysis

By studying the photoluminescence (PL) of the photoactive perovskite layer, we can assess the impact of the POMs on the non-radiative decay channels. The steady state PL spectra of the perovskite films spin-coated on the SnO_2_, SnO_2_/B1-W, and SnO_2_/B2-W are shown in Fig. 4B. An enhancement in the PL intensity of the SnO_2_/B1-W/perovskite samples compared with the control sample (SnO_2_/perovskite) is observed, which is even higher in the case of SnO_2_/B2-W/perovskite sample. This PL increase suggests less non-radiative recombination reaction of the perovskite film deposited on B1–W or B2–W, and therefore less energy losses.

The charge recombination kinetics can be analysed through time-resolved photoluminescence (TRPL), and provides information on the lifetime of electrons and holes in the perovskite layer. Usually, TRPL decays is fitted with a sum of exponential decays ${A_{ie}}^{{ - t/\tau }}i$ using Eq. (1), where (${A_i}\,{\mathrm{and}}\,{\tau _{\mathrm{i}}}$ are respectively the amplitude and lifetime of component* i*).

1 $$\:\boldsymbol{I}\boldsymbol{T}\boldsymbol{R}\boldsymbol{P}\boldsymbol{L}\:\left(\boldsymbol{t}\right)=\:{\sum\:}_{\boldsymbol{i}}^{\boldsymbol{n}}{\boldsymbol{A}}_{\boldsymbol{i}}\boldsymbol{*}\:{\boldsymbol{e}}^{-\frac{\boldsymbol{t}}{{\boldsymbol{\tau\:}}_{\boldsymbol{i}}}}\:$$

When the Eq. (1) is applied, it becomes a bi-exponential (*n* = 2) fit and the two components are usually assigned to trap-mediated recombination (shorter lifetime $\tau _{i}$ and radiative recombination (longer lifetime ${\tau _2}$ ) as shown in Eq. (2). The longer ${\tau _1}\,and\,{\tau _2}$ are, the more the charge recombination is reduced. The amplitude of each exponential component is attributed to the contribution to each process.

2 $$\:\boldsymbol{I}\boldsymbol{T}\boldsymbol{R}\boldsymbol{P}\boldsymbol{L}\:\left(\boldsymbol{t}\right)=\:\boldsymbol{A}1\boldsymbol{*}\:{\boldsymbol{e}}^{-\frac{\boldsymbol{x}}{\boldsymbol{\tau\:}1}}+\boldsymbol{A}2\boldsymbol{*}\:{\boldsymbol{e}}^{-\frac{\boldsymbol{x}}{\boldsymbol{\tau\:}2}}+{\boldsymbol{I}\boldsymbol{T}\boldsymbol{R}\boldsymbol{P}\boldsymbol{L}}_{\left(\boldsymbol{t}=\:0\right)}\:$$

The TRPL spectra presented in Fig. 4C show that all the perovskite peaks rise intensively after excitation followed by an exponential decay. Modified films (B1-W/perovskite and B2-W/perovskite) have a longer average lifetime with respect to the control one (Table 5). Hence, the incorporation of POMs suppresses the non-radiative charge recombination by possible decreasing the defects in the perovskite film and so contributes to enhancement of their performance.

**Table 5:** Fitting lifetime results parameters of TRPL spectra of perovskite films deposited on pristine, B1-W- and B2-W-modified SnO_2_ substrates.

Equation	Control	B1–W	B2–W
	$$ITRPL~\left( t \right) = A1*~e^{{ - \frac{t}{{\tau 1}}}} + A2*~e^{{ - \frac{t}{{\tau 2}}}} + ITRPL_{{\left( {t = 0} \right)}}$$		
τ1 (ns)	52.245	53.629	58.213
τ2 (ns)	235.04	302.214	279.73

**Fig. 5:**
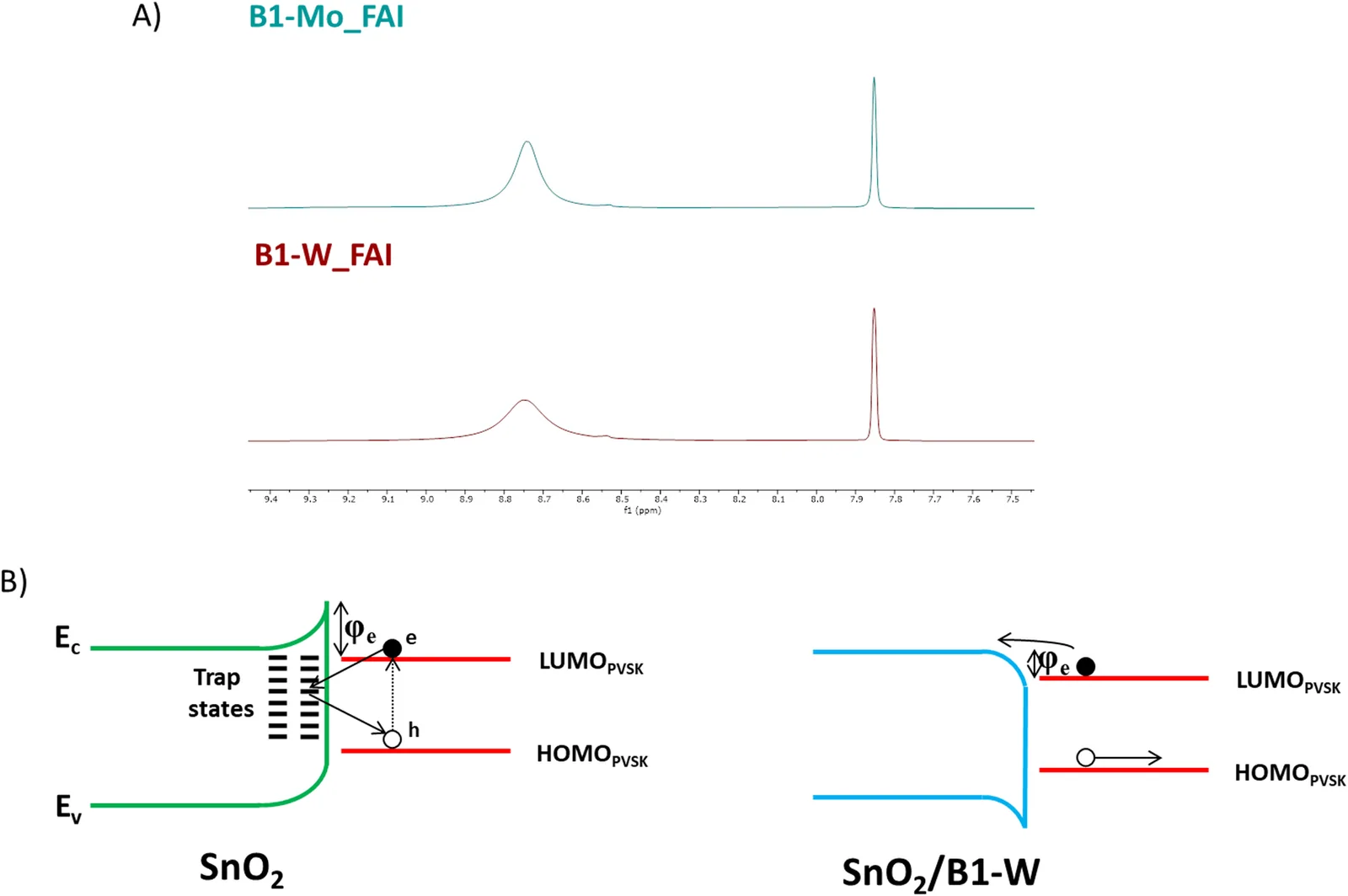
(**A**) Comparison of the^1^ H NMR spectra of B1-W and B1-Mo with FAI. (**B**) Schematic representation of reduced electron extraction barrier of the photo-generated charge carriers in the PSCs based on the POM B1-W.

To further investigate the passivation effect, the^1^ HNMR spectra of the FAI/B1-W, FAI/B1-Mo, PbI_2_/B1-W, and PbI_2_/B1-Mo mixtures in DMSO-*d*_6_ were recorded. Figure 5A shows the^1^ HNMR spectra of FAI with the addition of POM B1-W and B1-Mo. The two samples exhibit similar spectra, with the N–H protons of formamidinium iodide (FAI) resonating at 8.75 ppm (4 H) and the C–H one at 7.85 ppm (1 H). In the case of FAI/B1-W, the N–H resonance is significantly broadened compared with that of the FAI/B1-Mo sample, suggesting increased proton exchange in FA^+^/B1-W and a stronger interaction between FAI and B1-W. Moreover, the PbI_2_/B1-W and PbI_2_/B1-Mo spectra do not exert any proton signals, as shown in Figure S10. These spectra only contain the DMSO-*d*_6_ residual peak at 2.50 ppm and the water peak at 3.33 ppm.

The interaction between the POMs and the perovskite was also confirmed by Fourier transform infrared (FTIR) spectroscopy. Figure S11 shows the FTIR transmittance spectra of the perovskite films deposited on pristine and POM-modified SnO_2_ substrates. Peaks at 3400–3250 cm^-1^ correspond to the asymmetric and symmetric stretching vibrational mode of N–H, which are more pronounced in the B1-Mo/PVSK, B2-W/PVSK, and B2-Mo/PVSK samples. The peaks at 1714 cm^-1^ and 1352 cm^-1^ refer to stretching mode of the C = N and bending mode of the C–H bond of formamidinium, respectively, while the peak at 1046 cm^-1^ corresponds to the rocking mode of methylammonium. Moreover, NH_3_ scissoring, NH_3_^+^ bending and CH_3_-NH_3_^+^ rocking of methylammonium are located at 1615 cm^-1^, 1473 cm^-1^, and 909 cm^-1^, respectively. In the case of perovskite films deposited on B1-Mo, B2-W, and B2-Mo, the peak of C = N bond is more intense, suggesting that the bond is significantly elongated. In addition, all FTIR peaks of the perovskite films deposited on POMs are slightly shifted to lower wavenumbers suggesting the interaction of POMs with the underlying PVSK layer.

## Discussion

This study demonstrates that the introduction of POM-modified SnO₂ interlayers significantly enhances the performance and stability of perovskite solar cells. Devices incorporating B1- and B2-modified SnO_2_ interlayers exhibited higher efficiencies than the control device based on as-deposited SnO_2_, with the B1–W-modified PSC achieving the highest PCE value, best balance of charge extraction, recombination suppression, and highest stability. TPV and TPC analyses confirmed that B1-modified SnO_2_ provided the longest charge carrier lifetimes and the most efficient charge collection, leading to superior photovoltaic performance. Stability assessments further revealed that B–W- and B1–Mo-based devices maintained higher PCEs over time, while B2-based PCSs, despite initial efficiency gains, exhibited increased hysteresis and faster degradation. Work function measurements established that POMs effectively modulated energy levels, optimizing charge transport and interface interactions. The efficient passivation effect of SnO_2_ through deposition of POMs is demonstrated (Fig. 5B), which resulted in suppressed charge recombination, enhanced electron extraction, and thus, improved PSC performance. It is suggested that the downward band bending of the POM-modified SnO_2_, and especially of the SnO_2_/B1-W reduces the electron extraction barrier (φ_e_), and hence, enhances the electron extraction. Furthermore, the probability of the photo-generated electrons, that should be transferred from the LUMO of the perovskite layer to the valence band of SnO_2,_ to be trapped in the trap states of SnO_2_, which are usually localized in its band gap, and then recombine with the holes present in the perovskite’s HOMO is quite large. When the SnO_2_ surface is modified with the deposition of B1-W, the surface traps are suppressed hindering the charge recombination at the SnO_2_/B1-W/PVSK interface, resulting in the enhancement of the photocurrent, as well as the stability improvement of the PSC based on the B1-W-modified SnO_2_ EEL.

Morphological characterization confirmed that POMs B1–W and B2–W contributed to improved perovskite crystallization, facilitating larger grain formation and reducing defect-related recombination. PL and TRPL studies corroborated these findings, indicating enhanced charge carrier dynamics and minimized energy losses in POM-modified films. Overall, the results underscore the critical role of interfacial engineering in PSCs, with POM-based modifications offering a viable approach for improving device efficiency, stability, and commercial viability.

## Methods

### Device fabrication

All ITO substrates (Yingkou Shangsheng) were ultrasonic cleaned sequentially in deionized (DI) water and ethanol for 15 min. After 15 min UV-Ozone surface treatment, SnO_2_ was deposited onto clean ITO substrates by spin coating a 1:6 diluted SnO_2_ nanoparticle water solution (Alfa Aeser) at 4,000 rpm for 30 s, followed by annealing at 150°C for 15 min in air. Polyoxometalates (POMs) were dissolved by DI water with concentration of 0.5 mg/mL and the solutions were spin coated onto SnO_2_ at 2,000 rpm for 40 s, without further annealing process. Next, the perovskite films spin-coated on top of them. For the preparation of perovskite films, 188.8 mg of PbI_2_ (TCI), 63.8 mg of FAI (Greatcell Solar), 8.8 mg of MACl (Greatcell Solar), and 1 mg of RuCl (Sigma-Aldrich) were dissolved together with 200 µL of N, N’-dimethyl formamide (DMF, 99.8% anhydrous, Sigma-Aldrich) and 25 µL of dimethyl sulfoxide (DMSO, > 99.5%, Sigma-Aldrich). 50.5 mg of CsI (Sigma-Aldrich) were dissolved in 129 µL of DMSO. Then 5.68 µL of CsI solution is added into the precursor solution. The perovskite solution was spin-coated at 5,000 rpm for 30 s, during which 90 µL of chlorobenzene was quickly dropped at 12 s after spin-coating process started. Then, the perovskite films were annealed at 150 °C for 15 min. N-octylammonium iodide (OAI) solution (3 mg/mL in IPA) was spin-coated onto the perovskite film with 5,000 rpm for 20 s, followed by the annealing process at 100 °C for 3 min. Radical-doped spiro-OMeTAD (2,2′,7,7′-tetrakis(N, N-di-p-methoxyphenyl-amine)9,9′-spirobifluorene) solution was prepared by mixing 95 µL of spiro-OMeTAD solution (90 mg/mL in the chlorobenzene) with 5 µL of spiro-OMeTAD^2+^(TFSI^−^)_2_ (110 mg/mL in tetrachloroethylene) and 28 µL of TBMP^+^TFSI^−^ (110 mg/mL in tetrachloroethylene). The doped-spiro-OMeTAD solution was spin-coated at5,000 rpm for 20 s. No further annealing was needed. Finally, 50 nm of Au electrode was thermal-evaporated at 5 ×10^-6^ Torr to fabricate the full device. A 0.06 cm^2^ shadow mask was used to define the effective working area of the solar cells.

### Thin film and device characterization

Contact potential difference (CPD) was measured by the vibrating gold alloy Kelvin probe (2 mm). Absolute W_F_ of the tip was estimated to be around 4.54 ± 0.06 eV, which was calibrated by measuring a silver reference and calculating its absolute W_F_ by APS. Atomic force microscopy (AFM) images were taken using an XE7 microscope (Park Systems) operating in tapping mode. UV/Vis absorbance spectra were recorded using UV-1900i Shimadzu spectrometer. X-ray diffraction (XRD) patterns were analyzed on an X-ray diffraction meter (Panalytical, X’Pert) with a Cu Kα radiation source and a Smart Lab Rigaku diffractometer (θ/θ scan) with a CuKA radiation (3 kW). Scanning electron microscopy (SEM) images were recorded using a Zeiss Sigma 300 under 3 kV and a JEOL 7401f FESEM microscope. The time-resolved photoluminescence (TRPL) spectra were measured on an Edinburgh Instruments FLS1000 spectrometer with a 635 nm pulse laser. The steady photoluminescence (PL) spectra were obtained on the same instrument with a xenon lamp. FTIR spectra were recorded using a Bruker Tensor 27 spectrometer having a DTGS detector operating in a transmittance mode. ^1^H-NMR experiments were carried out on a Bruker Avance 400 MHz Ultrashield spectrometer, at 298 K, using the residual solvent peak as reference (DMSO-*d*_6_: δΗ = 2.50 ppm). Chemical shifts are given in ppm. ^1^H-NMR spectra of the samples were obtained at a concentration of 0.5 M in DMSO-*d*_6_ and loaded in 5 mm NMR tubes. J-V curves were measured (Keithley, 2400 Source Meter) under simulated AM 1.5 sunlight (100 mW cm^-2^, Enlitech AAA sun simulator) with the intensity calibrated by a Si reference cell.

### Grain size statistics

*Method 1: Watershed-based detection of grains and calculation of the boundary length to whole area ratio.* Image segmentation is performed using refined watershed-based segmentation. Initially, each image is read and converted to grayscale to simplify processing. Contrast enhancement is achieved through adaptive histogram equalization. Subsequently, the image is binarized using adaptive thresholding to distinguish foreground objects from the background, with noise reduction applied by eliminating small isolated objects and filling holes in the binarized image. The distance transform is then computed on the binary image and shallow minima suppression is applied. The watershed transform is finally employed on the modified distance image to delineate object boundaries. *Method 2: Correlation-length-based estimation of mean grain size and related boundary length.* This method is based on two assumptions. The first is that the relative length of grain boundaries is monotonically related to the density of grains. Smaller grain sizes (i.e. higher densities) are associated with larger boundary lengths and vice versa. The second is that the mean grain size is strongly linked to and can be quantified by the correlation length of grainy surfaces. To exploit these assumptions, we calculate the Height-Height Correlation Function of the AFM image and then we estimate the properly defined correlation length to characterize the grainy structures. The obtained correlation length is inversely related to the grain boundary length and therefore, smaller correlation lengths are expected in cases where the first method delivers large relative boundary lengths.

## Supplementary Information

Below is the link to the electronic supplementary material.


Supplementary Material 1.


## Data Availability

Data is provided within the manuscript or supplementary information files. The datasets used during the current study are also available from the corresponding author on reasonable request.
