# Supplementary material for: Lacunary polyoxometalate nanoclusters as versatile interface modifiers for perovskite solar cells
Source: Sci Rep. 2026 Sep 1;16:27313. doi: 10.1038/s41598-026-46107-7 (PMC13530181; doi:10.1038/s41598-026-46107-7)
Supplement: Supplementary file 1 [file 41598_2026_46107_MOESM1_ESM.docx]

**Supporting Information**

Lacunary Polyoxometalate Nanoclusters as Versatile Interface Modifiers for Perovskite Solar Cells

# **Apostolis Verykios^1^, Yasemin Torlak^2^, Fangwen Cheng^3^, Mélissa Crespin^3^, Marinos Tountas^4^, Nikolaos Tzoganakis^4^, Dimitrios K. Giannopoulos^5^, Antigoni Cheilari^5^, Georgios C. Vougioukalakis^5^, Ermioni Polydorou^1^, Zoi Georgiopoulou^1,9^, Georgios Chatzigiannakis^1^, Ioannis G. Aviziotis^1,6^, Vassilis Psycharis^1^, Efi – Maria Papia^1^, Vassilios Constantoudis^1^, Anastasia Soultati^1,4*^, Mahmut Kus^7^, Mustafa Ersoz^8^, Emmanuel Kymakis^4^, Alexander Chroneos^9,10^, Ioannis Karatasios^1^, Feng Gao^3^, Feng Wang^3,*^, Maria Vasilopoulou^1,*^**

^1^Institute of Nanoscience and Nanotechnology, National Center for Scientific Research Demokritos, 15310, Agia Paraskevi, Attiki, Greece

^2^Cal Vocational High School, Pamukkale University, 20700, Denizli, Turkey

^3^Department of Physics, Chemistry and Biology, Linköping University, Linköping, 58183, Sweden

^4^Department of Electrical & Computer Engineering, Hellenic Mediterranean University, Estavromenos, Heraklion, GR-71410, Crete, Greece

^5^Laboratory of Organic Chemistry, Department of Chemistry, National and Kapodistrian University of Athens, Panepistimiopolis, Athens, 15771, Greece

^6^School of Chemical Engineering, National Technical University of Athens, Heroon Polytechneiou 9, Zografou Campus 15771, Greece

^7^Department of Chemical Engineering, Konya Technical University, 42075, Konya, Turkey

^8^Department of Chemistry, Selcuk University, Konya 42250, Turkey

^9^Department of Electrical and Computer Engineering, University of Thessaly, Volos 38221, Greece

^10^Department of Materials, Imperial College London, London SW7 2AZ, United Kingdom

**Figure S1.** Transient A) photovoltage and B) photocurrent measurements of the pristine and the modified with POMs.

**Figure S2.** J-V characteristic curves of PSC using pristine, DI water-treated, and B1-W-modified SnO_2_.

**Figure S3.** UV-Vis absorption spectra of the four POMs films spin coated on quartz substrates.

**Figure S4.** A) Tauc plots and B) UV-Vis absorbance spectra of pristine and POM-modified SnO_2_ films.

**Figure S5.** 2D atomic force microscopy (AFM) topographies (5x5 μm^2^), along with the surface roughness measurements of the perovskite film taken on the as-deposited SnO_2_ and the POM-modified SnO_2_ substrates.

| **Control** | |
| --- | --- |
|  | |
|  |  |
| **B1-W** | **B1-Mo** |
|  |  |
|  |  |
| **B2-W** | **B2-Mo** |
|  |  |

**Figure S6.** Imaged of the grain structure and boundary characteristics of the perovskite films deposited on the pristine and the POM-modified SnO_2_ substrates.

**Figure S7.** A) Watershed-based detection of grains and calculation of the Boundary to whole area ratio. B) Correlation-length-based estimation of mean grain size and related boundary length.

**Figure S8.** Top view SEM images of perovskite films deposited on A) pristine, B) DI water treated and C) B1-W-modified SnO_2_ substrates.

**Figure S9.** XRD pattern of perovskite films deposited on SnO_2_ and DI water treated-SnO_2_ substrates.

**Figure S10.** Comparison of the ^1^H NMR spectra of B1-W and B1-Mo with PbI_2_.

**Figure S11.** FTIR transmittance spectra of perovskite films deposited on pristine and POM-modified SnO_2_ substrates.
